# Spontaneous regression of adenocarcinoma of submandibular gland

**DOI:** 10.1016/j.bjorl.2020.10.014

**Published:** 2020-11-22

**Authors:** Otávio A. Curioni, Pedro de Andrade Filho, Andreza de Jesus Prates, Abrão Rapoport, Rogério Aparecido Dedivitis

**Affiliations:** a Heliópolis Hospital, Departamento de Cirurgia de Cabeça e Pescoço/Otorrinolaringologia, São Paulo, SP, Brazil; b Loma Linda University Medical Center, Department of Otolaryngology/Head and Neck Surgery, CA, United States; c Universidade de São Paulo, Faculdade de Medicina, Departamento de Cirurgia de Cabeça e Pescoço, São Paulo, SP, Brazil

## Introduction

The disappearance of a cancer without a satisfactory explanation is rarely accepted in the medical setting. This process is called “regression” or “spontaneous remission” and, in exceptional cases, patients are cured of the disease. The formal definition as partial or complete disappearance of a malignant tumor in the absence of treatment or in the presence of inadequate therapy was named by Dr. Tilden Everson and Dr. Warren Cole in the 1960s.^1^

The incidence of spontaneous regression is estimated between 1 in 60,000 to 140,000 cases of cancer, although it is very difficult to define it based on clinical aspects.^2^

The medical literature is rich in case reports of malignancies confirmed by pathologic exam, with computed tomography (CT) or magnetic resonance imaging (MRI) showing generalized diseases with spontaneous regression, which covers almost all types of cancers and histology. Examples include some hematologic neoplasms, sarcoma, melanoma, neuroblastoma, astrocytoma, Merkel cell carcinoma and several locations of cancer (breast, lung, testicular, prostate, cervical, liver, colon, kidney).

In the head and neck, there are descriptions of spontaneous regression in neoplasms of oropharynx, tongue, mouth floor and larynx. In the case of the salivary gland, there are reports of regression from metastatic tumor of melanoma, Merkel cell carcinoma and regression of lung metastatic adenoid cystic of the parotid. The exact mechanism responsible for the remission is unknown.

## Case report

**Figure 1:**
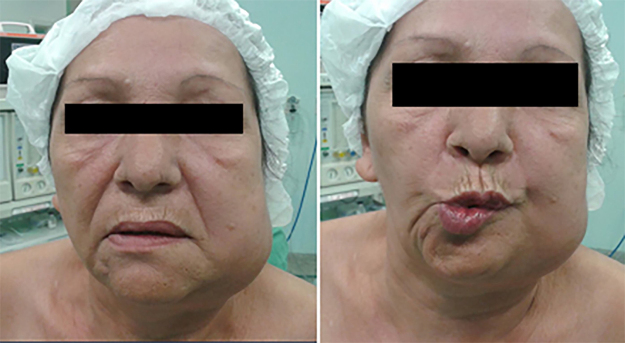
Clinical aspect at rest, evidencing facial asymmetry and paralysis of the marginal branch of VII cranial.

**Figure 2:**
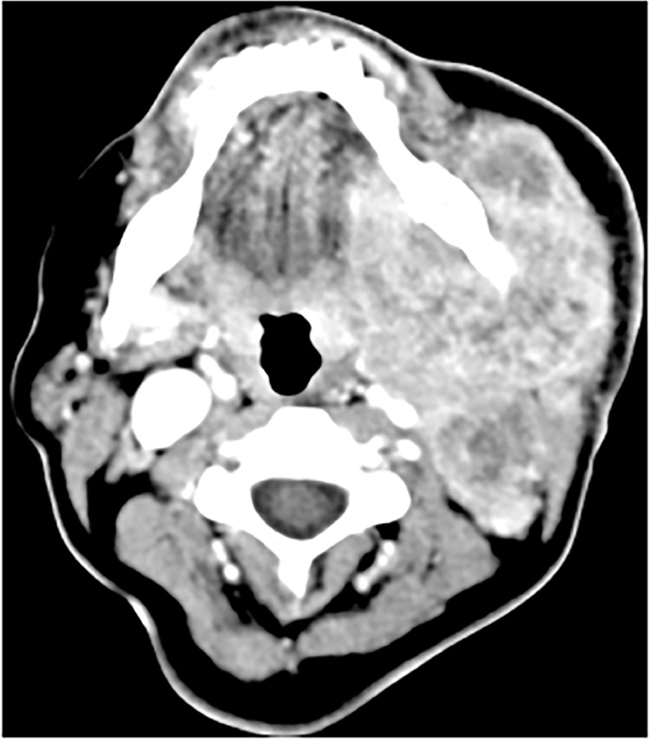
CT scan: Locally advanced left submandibular gland tumor.

**Figure 3:**
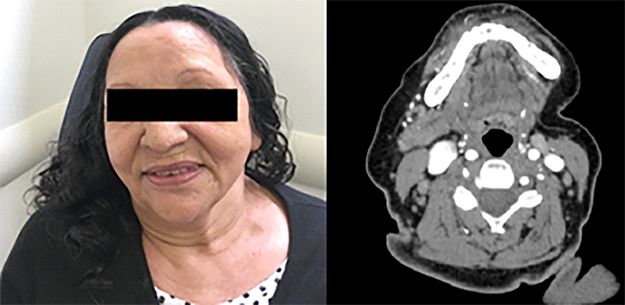
Absence of cervical tumorations 72 months after admission. CT scan maintained resolution of left submandibular gland tumor 75 months after remission.

A 51-year-old woman sought care at an outpatient clinic to evaluate a mass in the left submandibular region for the last 5 months, associated with worsening pain. There was previous history of smoking for 38 years. There was facial asymmetry with a 7 × 5 cm mass in the left submandibular gland, fixed to the mandibule, no lymph nodes palpable in the neck. The patient had paralysis of the marginal branch of the facial nerve (Fig. 1). Fine needle aspiration biopsy (FNAB) was suspicious for malignancy and CT scan showed a lesion in the left submandibular gland, involving the mandible without apparent bone invasion, and extensive necrotic tissue with infiltration of the floor of the mouth (Fig. 2). PET/CT showed a lesion involving the left submandibular gland (SUV mtox: 17.1) and cervical lymph nodes at level II, III, V on the left and bilateral IV level (SUV mtox: 10.7). An incisional biopsy was performed under local anesthesia and histologic sections showed an infiltrative poorly differentiated carcinoma; immunohistochemistry was positive for cytokeratin/CK-6 confirming epithelial differentiation. A diagnosis of low-grade adenocarcinoma was rendered. While waiting for surgical treatment, the patient presented signs of tumor regression and after 4 months the physical signs of the tumor completely disappeared. New imaging PET-CT tests were made and showed complete metabolic resolution of the lesion. The patient is undergoing outpatient monitoring without evidence of disease after 75 month follow-up (Fig. 3).

## Discussion

There are several cases of spontaneous resolution of malignant neoplasms, however, head and neck neoplasms that fit this definition are uncommon. We have not found reports of spontaneous regression of salivary gland adenocarcinoma in the Pubmed, VHL, CAPES and Cochrane research databases. There are descriptions of regression of melanoma metastases in salivary gland cancer,^3^ Merkel cell carcinoma^4^ and spontaneous regression of lung metastatic of parotid adenocystic carcinoma.^5^

The most accepted current theories for the triggering of spontaneous regression of tumors involve infectious processes and hypoglycemia as generators of exacerbated immunological response, being responsible for the healing mechanism. The theory of immunomodulation helped the medical understanding of spontaneous regression, which can be seen as the interaction of cancer with the host determining an incomplete elimination, a balance between forces or an escape of immune response.^6^

Under ideal conditions, the innate and adaptive elements of the immune system work together to eliminate cancer (often imperfectly) with regulatory (CD4+) and cytotoxic (CD8+) T cells, dendritic, natural killer cells (NK) and macrophages with a number of proteins secreted by immune activation, such as interferon gamma, interleukin 12 and tumor necrosis factor (TNF) working in harmony. Spontaneous regression may, in some cases, be a manifestation of this dynamic process.^7^

One of the keys to this process seems to be the stimulation of innate immunity. In series of reports of cases of spontaneous cure, the hypothesis was raised that the stimulus of an infectious condition helps the innate immune system to recognize tumor cells.^8^

Another theory under study that may corroborate the phenomena of spontaneous healing is the theory of hypoglycemia/hyperglycemia.^9^ This theory argues that a stimulus of external hypoglycemia, responded with gluconeogenesis and reactional hyperglycemia performs an activation of T cells.^10^

## Conclusions

This is an unusual event and the exact mechanism for such regression is unknown.

## Conflict of interest

The authors declare no conflicts of interest

## References

[1] Everson T.C. (1964). Spontaneous regression of cancer. Ann N Y Acad Sci.

[2] Chang W.Y. (2000). Complete spontaneous regression of cancer: four case reports, review of literature, and discussion of possible mechanisms involved. Hawaii Med J.

[3] King M., Spooner D., Rowlands D.C. (2001). Spontaneous regression of metastatic malignant melanoma of the parotid gland and neck lymph nodes: a case report and a review of the literature. Clin Oncol (R Coll Radiol).

[4] Mulder D.C., Rosenberg A.J., Storm-Bogaard P.W., Koole R. (2010). Spontaneous regression of advanced Merkel-cell-like small cell carcinoma of the parotid gland. Br J Oral Maxillofac Surg.

[5] Grillet B., Demedts M., Roelens J., Goddeeris P., Fossion E. (1984). Spontaneous regression of lung metastases of adenoid cystic carcinoma. Chest.

[6] Schreiber R.D., Old L.J., Smyth M.J. (2011). Cancer immunoediting: integrating immunity’s roles in cancer suppression and promotion. Science.

[7] Jhawar S.R., Thandoni A., Bommareddy P.K., Hassan S., Kohlhapp F.J., Goyal S. (2017). Oncolytic viruses-natural and genetically engineered cancer immunotherapies. Front Oncol.

[8] Niakan B. (2019). Common factors among some of the reported cases of the spontaneous remission and regression of cancer after acute infections. Int J Cancer Clin Res.

[9] Oiseth S.J., Aziz M.S. (2017). Cancer immunotherapy: a brief review of the history, possibilities, and challenges ahead. J Cancer Metastasis Treat.

[10] Oleksyszyn J., Wietrzyk J., Psurski M. (2014). Cancer – could it be cured? A spontaneous regression of cancer, cancer energy metabolism, hyperglycemia-hypoglycemia, metformin, Warburg and Crabtree effects and a new perspective in cancer treatment. J Cancer Sci Ther.

